# Unexpected Performance of a Bifunctional Sensitizer/Activator Component for Photon Energy Management via Upconversion

**DOI:** 10.1021/acs.jpclett.4c00720

**Published:** 2024-05-10

**Authors:** Giannis Antoniou, Stavros Athanasopoulos, Maria Koyioni, Panayiotis A. Koutentis, Panagiotis E. Keivanidis

**Affiliations:** † Device Technology and Chemical Physics Laboratory, Department of Mechanical Engineering and Materials Science and Engineering, Cyprus University of Technology, 45 Kitiou Kyprianou str., 3041 Limassol, Cyprus; ‡ Departamento de Física, Universidad Carlos III de Madrid, Avenida Universidad 30, 28911 Leganés, Madrid, Spain; § Department of Chemistry, University of Cyprus, P.O. Box 20537, 1678 Nicosia, Cyprus

## Abstract

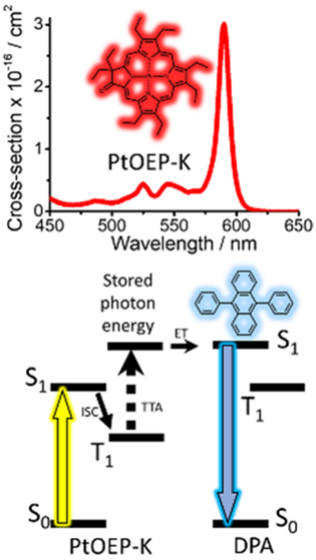

We here report on the observation of upconverted photoluminescence (UC-PL) from the blue-light-emitting 9,10-diphenylanthracene (DPA) mixed with the yellow-light-absorbing bifunctional sensitizer/activator component of (3,3,7,8,12,13,17,18-octaethylporphyrin-22,24-diid-2-one) Pt^II^ (PtOEP-K). Yellow-to-blue UC-PL (0.680 eV spectral upshift) is achieved at room temperature under ultralow power continuous incoherent photoexcitation (220 μW/cm^2^) despite the absence of triplet energy transfer (TET) between PtOEP-K and DPA. Under selective CW-laser photoexcitation of PtOEP-K in DPA:PtOEP-K, a 2.5% UC-PL quantum yield is obtained; that is an improvement exceeding by more than 3 orders of magnitude the UC-PL quantum yield of TTA-UC material combinations wherein no TET is operative. The PL response of DPA:PtOEP-K to varying laser fluence suggests that bimolecular annihilation reactions between triplet-excited PtOEP-K facilitate the UC-PL activation in DPA. These findings pave the way toward low-complexity strategies for the reduction of transmission losses in solar energy technologies through an innovative wavelength upshifting protocol involving excitonic materials.

Harnessing sunlight as an energy source can provide a sustainable supply for our energy-demanding societies.^1^ Solar-energy harvesting and storage device platforms such as photovoltaics (PV), photocatalysts and solar fuels^2,3^ hold promise to achieve this goal. However, these technologies face limitations due to transmission losses, which arise from their inability to use the entire spectrum of sunlight.^4,5^ Developing excitonic materials that possess triplet excitons capable of wavelength-shifting via photon energy upconversion can overcome this challenge.^6−8^ Due to extended lifetimes, triplet excitons can fuse via triplet–triplet annihilation (TTA) and activate a higher energy excited state that can power platforms for solar energy harnessing and storage. Especially for carbon-based excitonic materials, TTA-mediated photon energy upconversion (TTA-UC) photoluminescence (PL) has been observed for solution-processable organic dyes after direct laser photoexcitation to their first triplet (T_1_) excited state manifold (Figure 1a).^9^ Owing to the spin-forbidden nature of the S_0_ → T_1_ transition, TTA-UC PL emission is inefficient. However, incorporation of heavy atoms into the dye structure introduces strong spin–orbit coupling that circumvents the spin selection rule, leading to enhanced PL efficiency.^10^ This approach has yielded TTA-driven electron injection and photocurrent generation in dye-sensitized solar cells (DSSCs).^11^ A conventional method to achieve high TTA-UC efficiency uses binary sensitizer/activator systems (Figure 1b).^12^ In this scheme the sensitizer contains a heavy-atom that facilitates an efficient S_1_ → T_1_ intersystem crossing (ISC). Following the absorption of a low-energy photon through a spin-allowed S_0_ → S_1_ transition, the first T_1_ state of the sensitizer is efficiently populated via ISC. The stored electronic energy is then transferred to the T_1_ of an adjacent activator via a Dexter-type triplet energy transfer (TET) step. When the density of triplet-excited activators is increased, TTA reactions become operative leading to the activation of a higher-lying energy state capable of generating TTA-UC PL or activating a chemical reaction. In this process, the quantum yield for the preparation of a high-energy state is Φ_UC_ = Φ_ISC_ × Φ_TET_ × Φ_TTA_ ; where Φ_ISC_, Φ_TET_, and Φ_TTA_ correspond to the quantum yield of intersystem crossing in the sensitizer, triplet energy transfer from sensitizer to annihilator and TTA in the annihilator, respectively. Finally, a less explored excited state pathway for triplet fusion-induced upconversion involves the use a bifunctional single-component TTA-UC platform that has the capacity to strongly harvest the light, to undergo quantitative ISC and to activate a higher lying D* electronic state via TTA reactions (Figure 1c).^13−16^ Clearly, this is an attractive route to pursue since the use of a second component can be bypassed, thereby simplifying the process of TTA-UC-induced sensitization of photoactuating systems. The TTA-induced activation of the higher lying D* state in the sensitizer waives the need for a fast TET step from the sensitizer to an activator component. The elimination of the TET step minimizes photon energy losses and allows for the use of bifunctional sensitizer/activator species with relatively short phosphorescent lifetimes. To ensure the occurrence of TTA interactions, a high concentration of the sensitizer/activator species would be preferable. In this case the TTA-activated D* state is prepared with a quantum yield of Φ_UC_ = Φ_ISC_ × Φ_TTA_.

**Figure 1:**
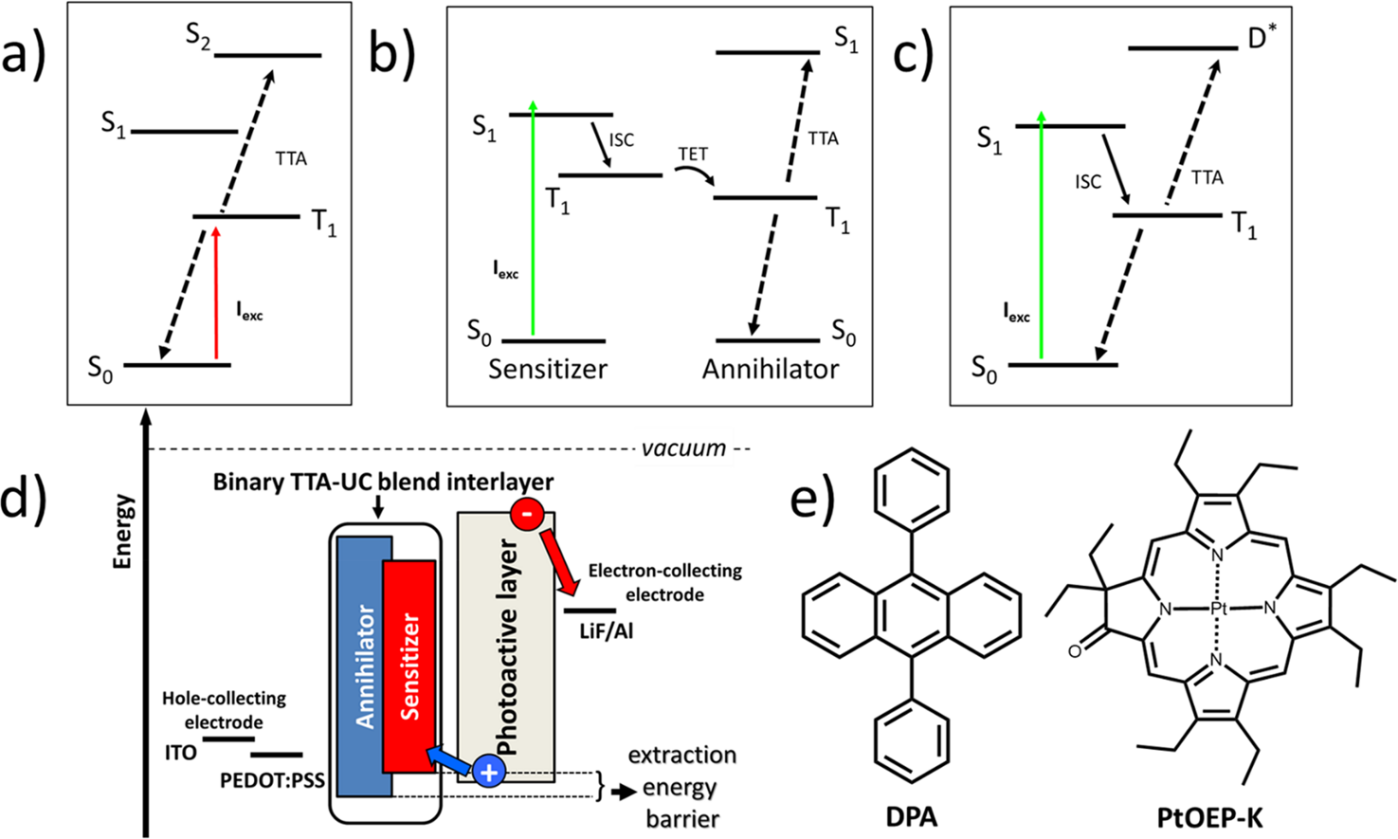
Low-photon energy-induced activation of a high energy electronic state by triplet–triplet annihilation (TTA) after (a) direct photoexcitation in the T_1_ state of an activator; (b) photoexcitation in the S_1_ state of a sensitizer followed by triplet energy transfer (TET) to the T_1_ state of an activator; and (c) photoexcitation in the S_1_ state of a bifunctional activator followed by intersystem crossing (ISC) to its T_1_ state. (d) Frontier energy level diagram of a binary TTA-UC interlayer electronically coupled to the photoactive layer of a PV device. ITO and PEDOT:PSS correspond to Indium Tin Oxide and poly(3,4-ethylenedioxythiophene) polystyrene sulfonate, respectively. (e) Chemical structures of DPA and PtOEP-K.

To this date, the conventional binary TTA-UC approach exhibits the highest reported TTA-UC performance reaching a TTA-UC PL quantum efficiency (PLQY_TTA-UC_) of 48% (relative to a theoretical maximum of 100%).^17^ Despite their high performance, conventional binary TTA-UC platforms in the solid state are incompatible with the established PV device geometries.^18,19^ Their electrical integration in devices with vertically stacked electrode configuration introduces more problems than the ones resolved by the TTA-UC-induced charge photogeneration. The desired optical and electrical integration of binary TTA-UC layers within the structure of PV devices results in the formation of energetic barriers that hinder efficient charge extraction (Figure 1d) and limit photocurrent generation.^19^ Due to the random distribution of the components in the binary TTA-UC layer and to their misaligned frontier orbitals in respect to the effective band gap of the photoactive layer and the work function of the device electrodes, no cascade energy level structure is established to facilitate efficient charge extraction. As such, the sensitization of charge photogeneration via TTA-UC is counter balanced by charge trapping and charge recombination losses.

To resolve this inherent constraint, bifunctional single-component TTA-UC materials (Figure 1c) are ideal to explore. It was shown recently how a single-component photoactive layer of (2, 3, 7, 8, 12, 13, 17, 18-octaethyl-porphyrinato) Pt^II^ (PtOEP), capable of TTA-driven photocurrent generation, offers the competitive advantage of serving simultaneously as photon absorber, triplet annihilator, activator for charge generation and charge extraction agent when embedded in an organic photodetector (OPD) device.^20^ Additional results corroborating the generic character of TTA-induced charge photogeneration were recently demonstrated^21^ for a carbazole-based molecular system that is typically used in organic light emitting diode (OLED) device platforms. In this regard, interlayers developed by single-component TTA-UC materials can be interfaced with active layers of light-harvesting systems within a device structure, by maintaining a cascade energy level alignment and without introducing charge extraction barriers.

To expand the portfolio of bifunctional TTA-UC material systems and to demonstrate their compatibility with commonly used emitters for TTA-UC applications, herein we present results on the unconventional material combination of 9,10-diphenylanthracene (DPA) mixed with (3,3,7,8,12,13,17,18-octaethylporphyrin-22,24-diid-2-one) Pt^II^ (PtOEP-K) (Figure 1e). DPA is the benchmark acene used as the annihilator/emitter component in binary TTA-UC systems,^22^ while PtOEP-K is an oxoclorin derivative^23^ that serves as an oxygen sensor component.^24^ The absorption and fluorescence spectra of DPA in toluene solution are presented in Figure 2a. The first spin-allowed S_0_ → S_1_ transition of DPA and its accompanied vibronic structure manifest in the 310–410 nm spectral range.^25^ The DPA fluorescence spectrum covers the 420–530 nm spectral region with a peak at 435 nm and with a 100% PL quantum yield.^25^

**Figure 2:**
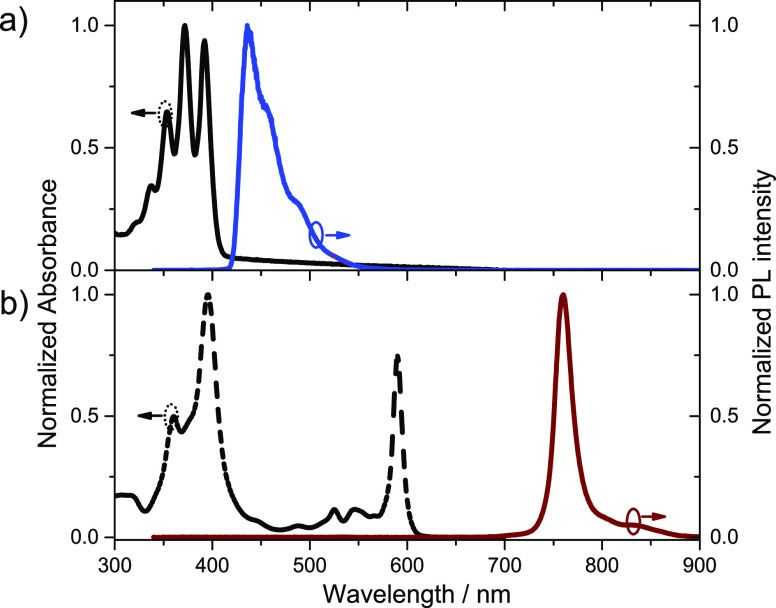
(a) Normalized absorbance (black solid line) and fluorescence (blue solid line, photoexcitation at 405 nm) spectra of a DPA solution in toluene (200 μM). (b) Normalized absorbance (black dashed line) and phosphorescence (brown solid line, photoexcitation at 532 nm) spectra of a PtOEP-K solution in toluene (150 μM). All spectra were collected at room temperature.

When in solution, PtOEP-K absorbs across the 330–610 nm spectral region with two characteristic bands centered at 395 and 590 nm (Figure 2b). These correspond to the Soret- and the Q-band of PtOEP-K, respectively.^23,24,26^ Time-dependent density functional theory (TD-DFT) calculations were further performed to compute the absolute vertical absorption energies of PtOEP-K and corresponding oscillator strength in toluene [see in Supporting Information (SI)]. In agreement with TD-DFT, the molar absorption coefficients (ε) associated with the Soret- and the Q-bands of PtOEP in toluene indicate their strongly allowed character with values of ε_395nm_ = 1.05 × 10^5^ M^–1^ cm^–1^ and ε_590nm_ = 7.86 × 10^4^ M^–1^ cm^–1^ (see in SI). With regards to the model PtOEP sensitizer typically employed in TTA-UC applications, the PtOEP-K derivative exhibits several attributes that are advantageous for enabling low-photon energy harvesting via TTA-UC. It absorbs low-frequency light stronger, has an absorption profile bathochromically shifted by ∼55 nm,^26^ and exhibits a marginally smaller spectral overlap between the DPA fluorescence and the PtOEP-K absorption. The Förster radius (*R*_0_) for resonance electronic energy transfer from singlet-photoexcited DPA to PtOEP is *R*_0_PtOEP_ = 38 Å, whereas for the DPA:PtOEP-K system it is *R*_0_PtOEP-K_ = 36 Å (see in SI). Due to the rapid ISC rate promoted by the Pt heavy atom, no measurable fluorescence is detected by the PtOEP-K derivative; the lifetime of the PtOEP-K phosphorescence is 60 μs with a phosphorescence PLQY of 12%.^23^ The PtOEP-K phosphorescence spectrum covers the 700–900 nm spectral range peaking at 760 nm, thereby placing the lowest triplet excited PtOEP-K state at 1.63 eV.

This is in very good agreement with excited state TD-DFT calculations that predict the lowest triplet emission energy of PtOEP-K in toluene at 757 nm, whereas the relaxed singlet state is expected at 566 nm. Table 1 presents an overview of the TD-DFT and PL characterization results for PtOEP-K. As such, the first triplet excited state of DPA^27^ is 180 meV higher in energy than the triplet excited state of PtOEP-K. Considering the thermal energy available at room temperature, the ∼7-fold difference corresponds to an energy barrier for triplet energy transfer from PtOEP-K to DPA. Nonetheless, the DPA:PtOEP-K system exhibits an intriguing TTA-UC PL response.

**Table 1:** TD-DFT Computed Triplet and Singlet Excited State Emission Energies (eV), Wavelengths (λ), and Oscillator Strength (*f*) of PtOEP-K in Toluene Obtained with the M11-L Exchange Correlation Functional and a Mixed 6-311++G(d,p)/Lanl2DZ Basis Set

transition	energy [eV]	λ [nm]	*f*	energy^a^ [eV]	λ^a^ [nm]
T_1_ → S_0_	1.6381	756.86		1.63	760
S_1_ → S_0_	2.1904	566.03	0.1913		

a Experimentally determined values from PL spectra of a PtOEP-K solution (150 μM) in toluene at room temperature.

Figure 3a presents the room-temperature PL spectra of a DPA:PtOEP-K solution in toluene (30 mM/150 μM), as registered after CW laser photoexcitation at 532 nm, with an increasing optical power density between 825 μW/cm^2^ and 47 W/cm^2^. In all cases the PtOEP-K phosphorescence signal is observed. Unexpectedly, the upconverted DPA luminescence is also detected for power densities higher than 44 mW/cm^2^. What’s more, after optimization of our PL detection system the registration of the upconverted DPA luminescence intensity is possible even when lower photoexcitation power densities are used. For these measurements extreme care was taken to ensure that no PtOEP residues were present in the PtOEP-K powder (see ^1^H NMR, MALDI-TOF, and TLC results in the SI). Moreover, the absence of PtOEP contamination in our DPA:PtOEP-K solutions was confirmed by comparitive PL measurements performed with laser photoexcitation at 532 nm and 590 nm (see SI).

**Figure 3:**
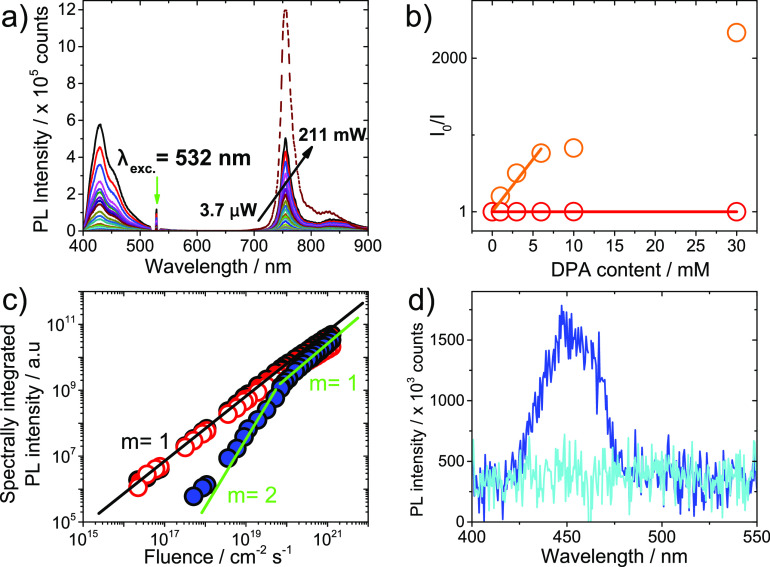
(a) Room-temperature PL spectra of DPA:PtOEP-K (solid lines) and PtOEP-K-only (dashed line) solutions after photoexcitation at 532 nm. (b) Phosphorescence quenching Stern–Volmer plots for DPA:PtOEP (orange circles) and DPA:PtOEP-K (red circles) solutions. Solid lines are linear fits on the data. (c) Fluence-dependent spectral integrals of PtOEP-K phosphorescence (open red circles) and DPA TTA-UC luminescence (filled blue circles) for a DPA:PtOEP-K solution (30 mM/150 μM), and of PtOEP-K phosphorescence (filled red circles) for a PtOEP-K reference solution (150 μM). Solid lines are guides to the eye. (d) Room temperature PL spectra of DPA:PtOEP-K (30 mM/150 μM, blue line) and DPA-only (30 mΜ, cyan line) solutions, selectively photoexcited with continuous incoherent light from the filtered output of a Hg–Xe Arc lamp (λ_exc_ = 600 ± 40 nm). All solutions were prepared in deaerated toluene.

The findings reported in Figure 3a are surprising given that no pulsed laser excitation was used in these measurements and despite the inability of PtOEP-K to sensitize the triplet level of DPA. For reference, at the highest excitation power a PtOEP-K-only solution in toluene (150 μM, 47 W/cm^2^) exhibits nearly two times stronger PtOEP-K phosphorescence than the corresponding one of the DPA:PtOEP-K system. However, the below described Stern–Volmer quenching analysis ruled out the occurrence of a Dexter energy transfer between the triplet-excited PtOEP-K and DPA.

For a set of DPA:PtOEP-K solutions with a PtOEP-K concentration fixed at 1.5 μM, the PtOEP-K phosphorescence was monitored after quasi-CW laser photoexcitation of 6.7 W/cm^2^ at 532 nm, where only PtOEP-K absorbs. The PtOEP-K phosphorescence intensity of these solutions remains unquenched as the DPA molar content increases gradually between 0 and 30 mM (Figure 3b).

In contrast, when the archetypical exothermic TTA-UC system of DPA:PtOEP is measured in an identical manner, the intensity of the PtOEP phosphorescence is quenched quantitatively already by 1 mM of DPA concentration, with a Stern–Volmer constant of K_SV_ = 1.36 × 10^5^ M^–1^. Therefore, in Figure 3a the observed quenching of PtOEP-K phosphorescence in the presence of a high DPA concentration (30 mM) is attributed to collisional interactions that are enhanced at high photoexcitation power.

Insight into the unexpected TTA-UC process can be gained by examining the photoexcitation intensity-dependent luminescence response of the DPA:PtOEP-K system. Based on the PL spectra (Figure 3a), the dependence of the spectrally integrated PtOEP-K phosphorescence and DPA TTA-UC PL intensity on fluence is constructed. Figure 3c displays the derived data in a double logarithmic plot. At low fluence (Φ) values, the TTA-UC PL intensity of DPA exhibits a nearly quadratic growth with Φ. However, beyond the critical value Φ_c_ = 5.3 × 10^19^ cm^–2^ s^–1^ (1.95 W/cm^2^), the dependence becomes linear. On the other hand, the PtOEP-K phosphorescence exhibits a linear scaling up to Φ_c_, beyond which it transitions to a sublinear trend. This linear-to-sublinear transition is also observed for the phosphorescence signal of the PtOEP-K-only reference solution, confirming the occurrence of TTA reactions PtOEP-K at high photoexcitation intensities.

In the high fluence regime, bimolecular TTA reactions between triplet-excited PtOEP-K (represented by the *k*_TTA_[*T*_1_]_PtOEP-K_^2^ kinetic term; see rate-equation kinetic model in SI) predominate over the monomolecular PtOEP-K deactivation (expressed by the rate constant *k*_*m*_).^28,29^ Under these conditions, TTA reactions in PtOEP-K activate a higher lying PtOEP-K electronic state that can transfer energy to DPA and generate the TTA-UC PL signal (Figure 1c). For the TTA-UC PL intensity of the DPA:PtOEP-K system, the transition from quadratic to linear dependence on fluence occurs at high photoexcitation intensities where *k*_m_ ≪ *k*_TTA_[*T*_1_]_PtOEP-K_ due to the increased density of the triplet-excited PtOEP-K molecules. In this photoexcitation regime, a PLQY_TTA-UC_ of 2.5% is obtained (relative to a theoretical maximum of 100%) when the DPA:PtOEP-K solution is photoexcited with Φ = 1.1 × 10^20^ cm^–2^ s^–1^ (4 W/cm^2^) of 532 nm CW-laser excitation. Despite the out of resonance photoexcitation wavelength used, the result demonstrates an improvement of at least 3 orders of magnitude compared to a previously presented poly(fluorene) (PF)/PdOEP system wherein a vanishing PLQY_TTA-UC_ of the PF emitter was obtained by TTA reactions in PdOEP.^13^

For real world solar energy harvesting and photocatalytic applications, the use of multifunctional single-component TTA-UC platforms requires the compatibility of TTA-UC performance with incoherent light sources. The DPA:PtOEP-K system meets this requirement as its yellow-to-blue TTA-UC luminescence can be activated under continuous incoherent photoexcitation. Figure 3d displays the TTA-UC PL spectrum of a DPA:PtOEP-K solution in toluene after photoexcitation by a Hg–Xe Arc lamp with an intensity of 220 μW/cm^2^. For this measurement, a combination of a band-pass and a long pass filter was used to reject the high photon energy portion of the Hg–Xe lamp output, thereby enabling selective photoexcitation of PtOEP-K across the 600 ± 40 nm spectral range. The generated TTA-UC luminescence was detected with an integration time of 30 s in the 450 ± 40 nm spectral window through a band-pass filter. As shown in Figure 3d, no measurable PL signal was obtained from a DPA-only solution that was photoexcited under identical photoexcitation conditions, thereby confirming the effective filtering of the Hg–Xe Arc lamp output during the measurement.

The noncentrosymmetric chemical structure of the metalorganic PtOEP-K complex may facilitate a second-order nonlinear absorption process and a sequential absorption route could be possible from the first triplet excited state to higher energy triplet PtOEP-K states. However, the occurrence of TTA-UC PL in DPA:PtOEP-K under weak continuous incoherent photoexcitation makes it unlikely that mechanisms involving second-order nonlinear absorption and reverse saturable absorption (RSA)^30,31^ are at play. Previous work on the study of rigid solid-state ternary organic blends for RSA^32^ showed that under low-intensity continuous incoherent white light, triplet excited state absorption requires highly diluted triplet excitons in the blend and extremely long-lived triplet exciton lifetimes (>1 s). For our work, the PtOEP-K system has a reported triplet excited state lifetime of 60 μs^23^ that is expected to be even shorter at the 150 μM concentration level of the studied DPA:PtOEP-K solutions. Moreover, the observed TTA-UC PL of DPA:PtOEP-K is in good agreement with previous reports on TTA-UC combinations of the centrosymmetric PtOEP.^33^ In those systems the generation of upconverted luminescence is not involving a TET step but it is facilitated by a TTA-activated metal-centered *d*–d* state in PtOEP.^34^ Recent work on PtOEP blended with the blue-light emitting poly(fluorene-2-octyl) (PFO) derivative proposed how TTA reactions in PtOEP activate the S_1_ state of PFO within the 100 fs time scale.^35^ Similarly, the TTA-induced activation of the Pt-centered state of PtOEP-K may sensitize the observed DPA TTA-UC luminescence (Figure 4a).

**Figure 4:**
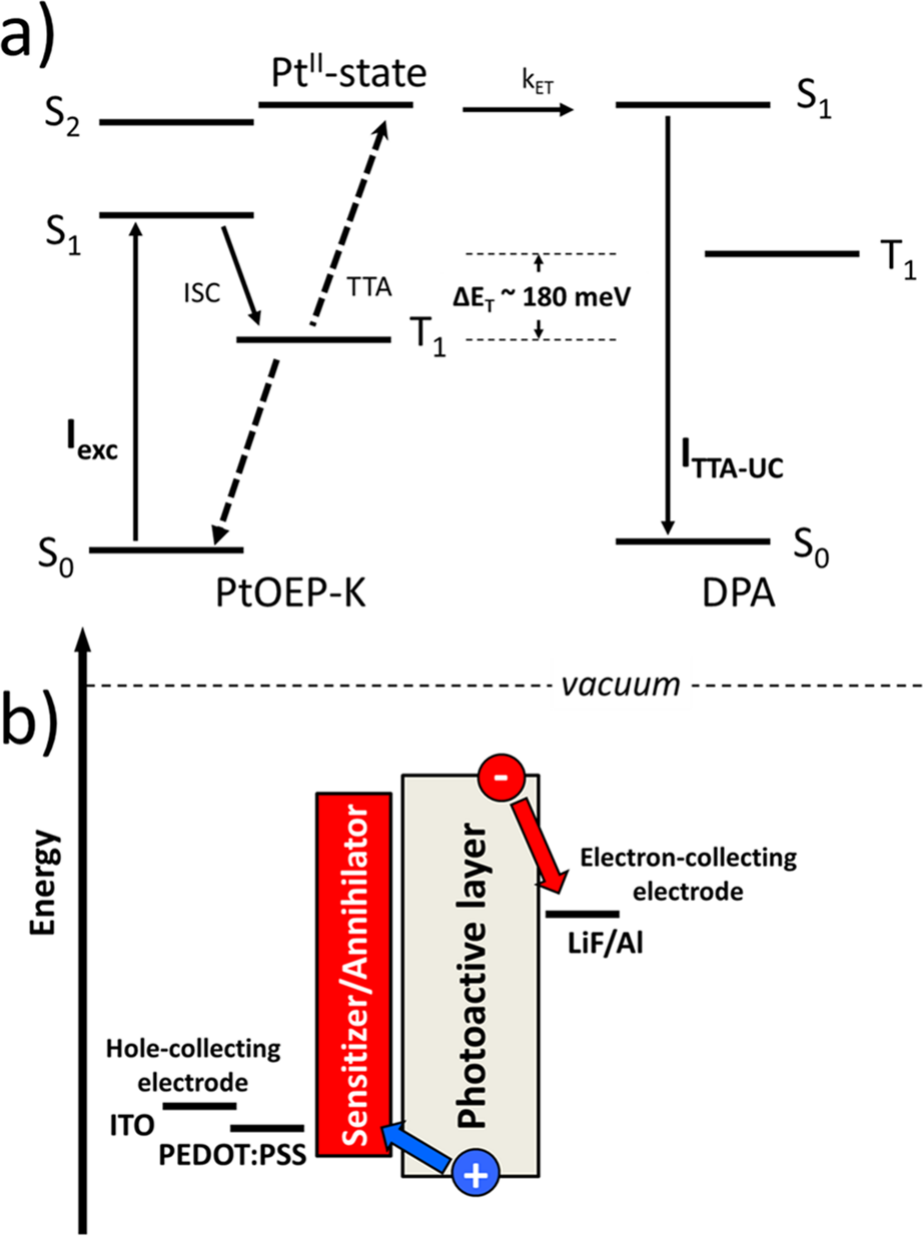
(a) Jablonski diagram illustrating the main photophysical processes in the DPA:PtOEP-K system that contribute to the generation of the DPA TTA-UC luminescence. (b) Frontier energy level diagram of a bifunctional single-component TTA-UC interlayer electronically coupled to the photoactive layer of a vertically stacked photodiode device.

By replacing DPA with another high energy gap photoactuator, photons with energy lower than the energy gap could support the photoactuation process. Figure 4b displays the concept of interfacing a PtOEP-K interlayer with the photoactive layer of a photodiode device and integrating it electrically within the vertically stacked electrode configuration. By transferring the photon energy stored in PtOEP-K via TTA to the photoactive layer, the sensitization of photocurrent generation with photon energies lower than the energy gap of the photoactive layer is possible without introducing detrimental charge extraction barriers (Figure 4b). As such, PtOEP-K and alike excitonic materials hold the potential to reduce transmission losses and to drive the sensitization of solar fuels,^4^ DSSCs,^36^ PV,^18^ and photocatalytic devices^37^ via wavelength upshifting. This is achievable in a simple manner (process displayed in Figure 1c), without the need to involve a second component (process displayed in Figure 1b).

In conclusion, we have studied the unexpected TTA-UC PL performance of the bifunctional PtOEP-K sensitizer/activator system when combined with the DPA emitter. Although the TTA-UC PL response of DPA:PtOEP-K is driven by TTA reactions between triplet-excited PtOEP-K, the obtained PLQY_TTA-UC_ is only an order of magnitude lower than that of conventional binary TTA-UC systems. Optimizing the optical excitation of PtOEP-K to coincide with the peak of its absorption Q-band could further narrow this gap and enable efficient photoexcitation of PtOEP-K with power densities closer to solar irradiance. The breakthrough achievement in the TTA-UC performance level of DPA:PtOEP-K strengthens the concept of employing single-component bifunctional TTA-UC materials for photon energy management. Previously confined in the zone of academic curiosity, these upconversion systems now stand as disruptive components to enable a paradigm shift in the sensitization of solar energy technologies via wavelength upshifting. Detailed investigations on the time-dependent photophysical properties of the DPA:PtOEP-K system and the electrical integration of solid-state PtOEP-K layers into OPV and OPD devices, are now underway.

## Supporting Information Available

Experimental methods, TD-DFT calculation results, molar absorption coefficient spectra of PtOEP and PtOEP-K solution in toluene, spectral integrals of DPA:PtOEP and DPA:PtOEP-K solutions, ^1^H NMR spectra, MALDI-TOF mass spectra, TLC analysis results, time-integrated PL spectra under pulsed laser photoexcitation at 532 and 590 nm, and rate-equation kinetic model for the DPA:PtOEP-K system (PDF)
