# Supplementary material for: Unexpected Performance of a Bifunctional Sensitizer/Activator Component for Photon Energy Management via Upconversion
Source: J Phys Chem Lett. 2024 May 10;15(20):5337–43. doi: 10.1021/acs.jpclett.4c00720 (PMC11129295; doi:10.1021/acs.jpclett.4c00720)
Supplement: Supplementary file 1 — jz4c00720_si_001.pdf [file jz4c00720_si_001.pdf]

Supporting Information

Unexpected Performance of a Bifunctional Sensitizer/Activator Component for Photon Energy Management via Up-Conversion

Giannis Antoniou [1], Stavros Athanasopoulos [2], Maria Koyioni [3], Panayiotis A. Koutentis [3], Panagiotis E. Keivanidis [1,*]

[1] Device Technology and Chemical Physics Laboratory, Department of Mechanical Engineering and Materials Science and Engineering, Cyprus University of Technology, 45 Kitiou Kyprianou str., Limassol 3041, Cyprus

[2] Departamento de Física, Universidad Carlos III de Madrid, Avenida Universidad 30, 28911 Leganés, Madrid, Spain

[3] Department of Chemistry, University of Cyprus, P. O. Box 20537, 1678 Nicosia, Cyprus

KEYWORDS triplet-triplet annihilation, wavelength shifting, transmission losses, delayed fluorescence, singlet fission

Materials and methods

Materials and solution preparation: DPA was purchased from Sigma Aldrich whereas PtOEP-K and PtOEP were purchased from Frontier Scientific Inc. All materials were used as received without further purification and solutions were prepared in de-aerated toluene as the solvent. Degassing was achieved by purging the solvent with N_2 (5.0) for 45 min during ultrasonication. The purity of PtOEP-K was verified by ^{1}H NMR and MALDI-TOF spectra. ^{1}H NMR spectra were recorded on a Bruker Avance III 500 machine at 500 MHz. Deuterated chloroform was used for homonuclear lock, and the signals were referenced to the non-deuterated residual peak. MALDI-TOF mass spectra were recorded on a Bruker Autoflex III Smartbeam instrument.

UV-vis absorption characterization: Absorption spectra of solutions were recorded with a UV-2700 Shimadzu spectrophotometer operated in transmission mode. A high precision quartz glass cuvette of 1 mm path length was used and the obtained spectra were corrected with respect to the absorption spectrum of pure toluene. In all cases, a spectral band width of 1 nm and a scan rate of 1 nm/s were used.

Time-integrated photoluminescence (PL) characterization: Laser-induced PL spectra of the prepared solutions were recorded with a right-angle geometry at room temperature. The solutions were photoexcited either at $\lambda_{exc.}$= 405 nm with a laser diode (E0405-10SM, Edmund Optics) or at 532 nm with CW-diode-pumped solid-state (CW-DPSS) laser (MGL-III-532, CNI Optoelectronics Tech.CO. Ltd). The emitted light was dispersed in a mini spectrograph (FLAME-S-VIS-NIR-ES, Ocean Insight) and detected with a Sony ILX511B linear silicon CCD array. During PL detection the intensity of the excitation wavelength was suppressed by appropriate filters (NF405-13, Thorlabs for $\lambda_{exc.}$= 405 nm and NF01-532U, Semrock for $\lambda_{exc.}$= 532 nm). Photoexcitation intensity-dependent PL measurements were performed by using the combination of a set of neutral density filters of known transmittance values. Quasi-CW PL measurements were performed by modulating the output of the CW-DPSS 532 nm laser beam with a mechanical chopper (MC2000B, Thorlabs) at 10 Hz and with a 50% duty cycle (MC1F2 chopper blade, Thorlabs). The PL detection system was synchronized in respect to the modulated photoexcitation light by electrical triggering that was provided by a reference photodiode (SM05PD1A, Thorlabs) and a dedicated break out board

(FLAME-DD4-Breakoutboard, Ocean Insight). In all cases, the average laser power was determined with a thermopile power sensor (PS19Q, Coherent Inc.) coupled to a FieldMaxII-TOP power/energy meter (Coherent Inc.).

Time-integrated PL characterization under incoherent photoexcitation: A Hg/Xe light source (Research F/2.2 Illumination Source 200 W Hg/Xe Ozone-free Arc Lamp, Newport) was used for photoexciting at 600±40 nm. Care was taken to ensure the elimination of any parasitic photoexcitation of the DPA component by high photon energy residues of the lamp output, by rejecting the high photon energy portion of the Hg-Xe lamp output with a combination of a band pass (FB600-40, Thorlabs) and a long pass (FG550S, Thorlabs) filter. The photoexcitation intensity used was determined based on a calibrating photodiode (818-UV/DB UV Detector, Newport DB15 Calibration Module) with known responsivity. The generated luminescence was detected in the 450±40 nm spectral region through a band pass filter (FB450-40, Thorlabs). The PL spectra were collected with a Newton CCD camera (DU920P-BEX2, Oxford Instruments) coupled to a Czerny-Turner spectrograph (Kymera-328I-B1-SIL, 328 mm focal length, F/4.1 aperture, Oxford Instruments).

PL quantum yield (PLQY) determination: PLQY data were obtained by using a Rhodamine 6G (R6G) dilute solution in ethanol as a reference, after photoexcitation under 532 nm. The molarity of the R6G solution was kept low so that a measurable optical density lower than 0.05 was obtained at the photoexcitation wavelength. The UV-vis absorption and PL data of the reference solution were identically recorded as the DPA:PtOEP-K solutions in toluene. PLQY values were calculated via the following equation, whereby *Q*, *I*, *OD* and *n* represent quantum yield, PL intensity, optical density and solvent refractive index, respectively. Indicator *R* corresponds to the reference solution for which a Q_R of 94% was considered.

$$Q = Q_R \frac{I}{I_R} \frac{OD_R}{OD} \left(\frac{n}{n_R}\right)^2 \qquad \text{Eq. 1}$$

Density functional theory (DFT) calculations: DFT was used with the dual-range local exchange-correlation functional M11-L and a mixed basis set, the triple-ζ Pople 6-311++G(d,p) basis set for C, H, N and O atoms and a Lanl2DZ basis with effective core potential for Pt to obtain the ground state geometry

of PtOEP-K without imposing any symmetry constraints. Subsequently, singlet ($S_0 \rightarrow S_n$) and triplet ($S_0 \rightarrow T_n$) vertical excitation energies were computed from linear response time-dependent density functional theory (TD-DFT). Solvent effects on the electronic spectra are introduced using the polarisable continuum model with toluene as a solvent. The lowest energy singlet ($S_1 \rightarrow S_0$) and triplet ($T_1 \rightarrow S_0$) emission energies were computed by optimizing the corresponding S_1 and T_1 excited state geometries. Quantum chemical calculations were performed with the Gaussian 16 software. [1]

TD-DFT calculation results

	PtOEP-K		
**Transition**	**Energy / [eV]**	**λ / [nm]**	**f**
$S_0 \rightarrow T_1$	1.7384	713.23	-
$S_0 \rightarrow S_1$ (Q-band)	2.2522	550.49	0.1943
$S_0 \rightarrow S_2$ (Soret-band)	3.1130	398.28	0.4859

Table S1. TD-DFT computed lowest energy triplet and singlet (Q- and Sorer- band) excited state absorption energies (eV), wavelengths (λ) and oscillator strengths (f) of PtOEP-K in toluene obtained with the M11-L exchange correlation functional and a mixed 6-311++G(d,p)/Lanl2DZ basis set.

Molar absorption coefficient spectra

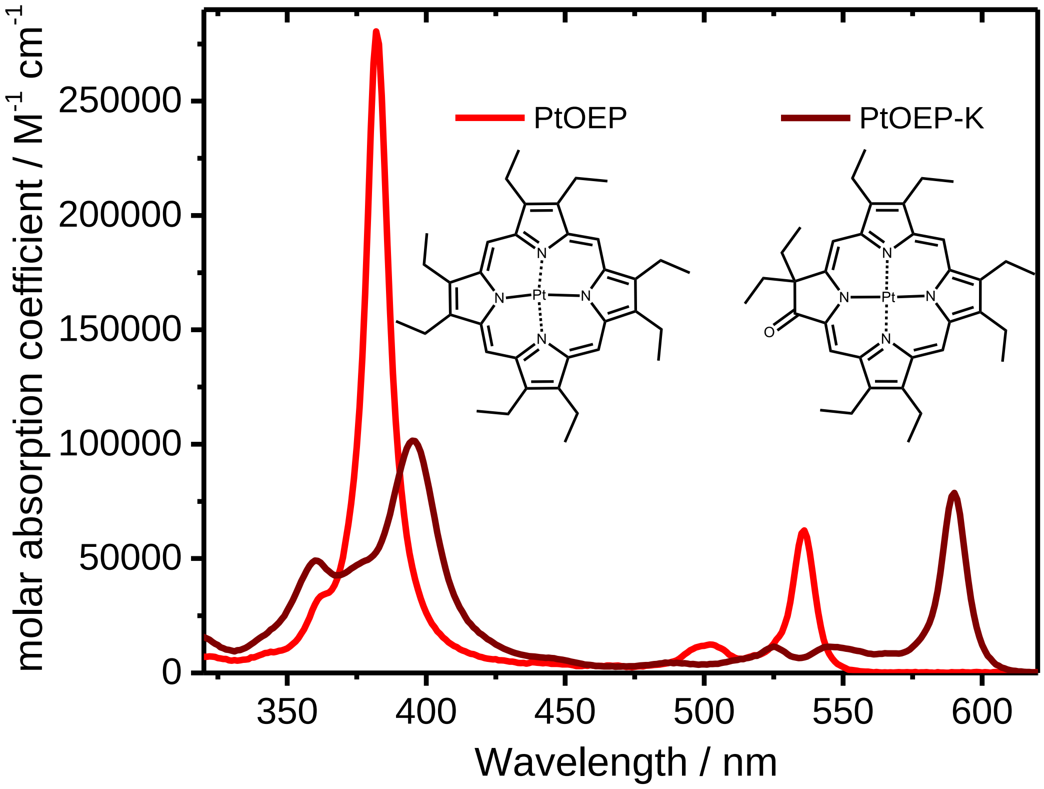

Figure S1. Molar absorption coefficient spectra of PtOEP (red line) and PtOEP-K (brown line) in toluene solutions.

Spectral integrals

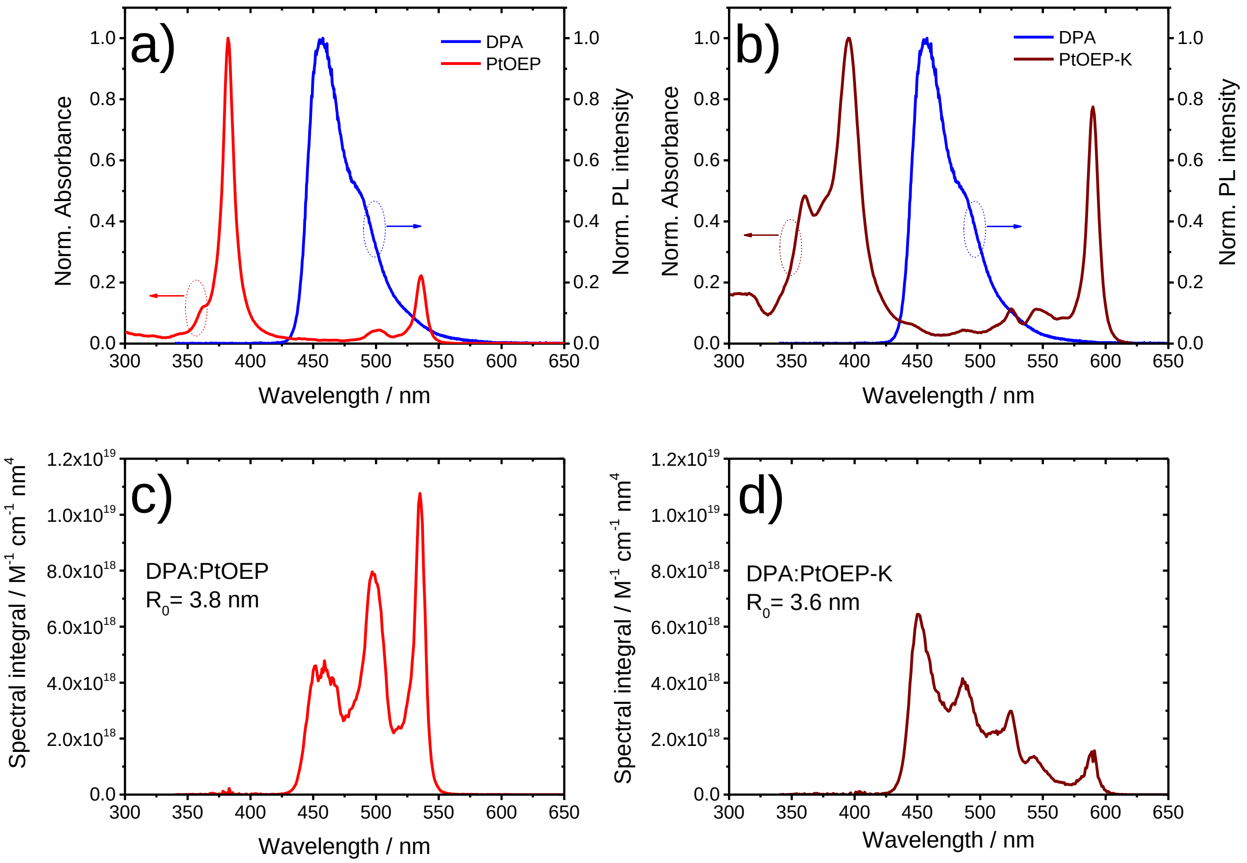

Figure S2. Normalized room temperature spectra of a) PtOEP absorbance (red line) and DPA fluorescence (blue line), b) PtOEP-K absorbance (brown line) and DPA fluorescence (blue line). The corresponding spectral integrals of c) DPA:PtOEP and d) DPA:PtOEP-K.

^{1}H NMR results

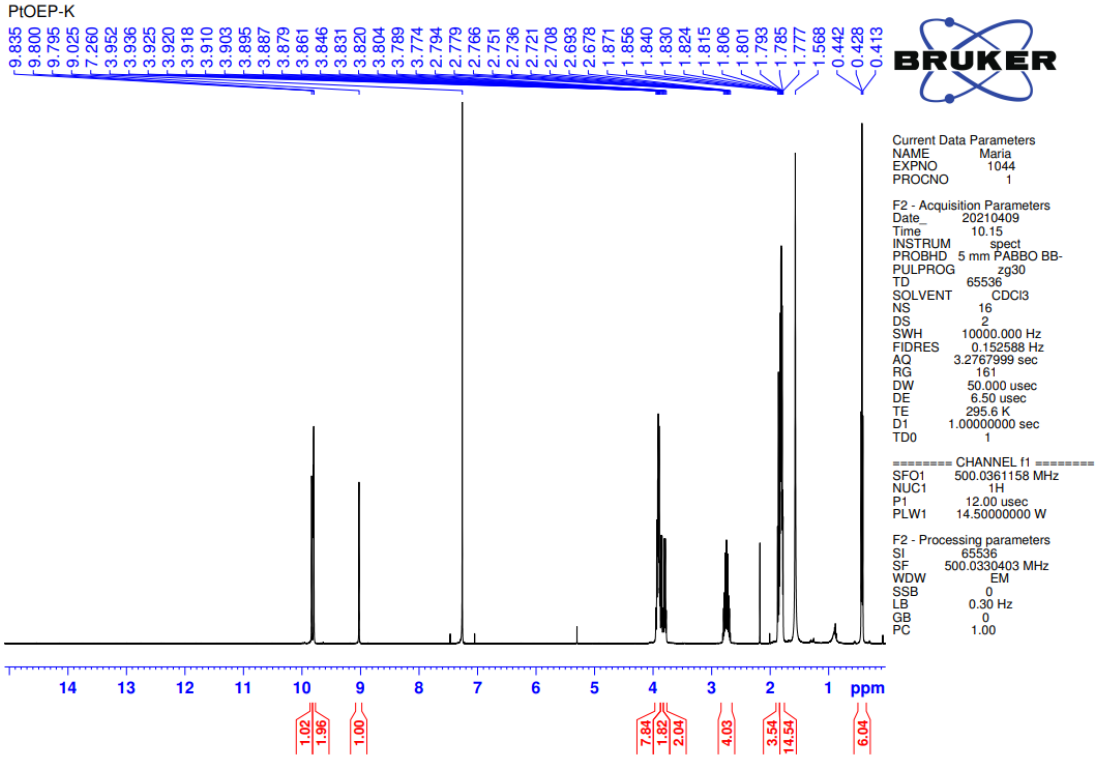

Figure S3. ^{1}H NMR spectra of PtOEP-K solution in $CDCl_3$: δ_H(500 MHz; CDCl3) 9.83 (1H, s, CH), 9.80 (1H, s, CH), 9.80 (1H, s, CH), 9.02 (1H, s, CH), 3.95-3.88 (8H, m, 4 × CH_2), 3.85 (2H, q, *J* 8.1 Hz, 1 × CH_2), 3.80 (2H, q, *J* 7.7 Hz, 1 × CH_2), 2.81-2.68 (4H, m, 4 × CH_2), 1.86 (3H, t, *J* 7.8 Hz, 1 × CH_3), 1.83-1.78 (15H, m, 5 × CH_3), 0.43 (6H, t, *J* 7.4 Hz, 2 × CH_3).

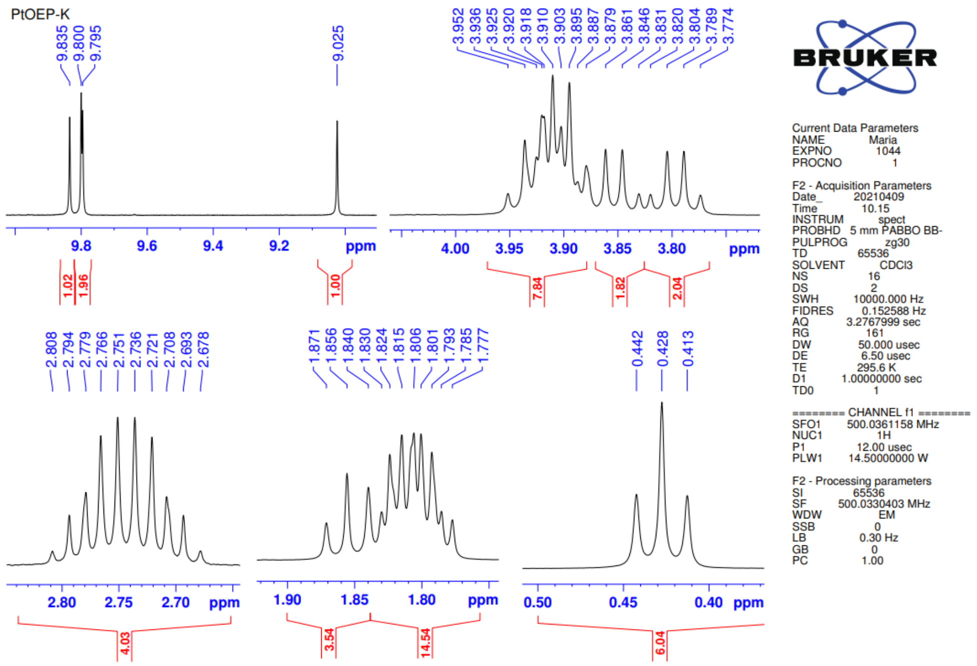

Figure S4. Downfield expansion of the ^1H NMR spectra presented in Figure S1.

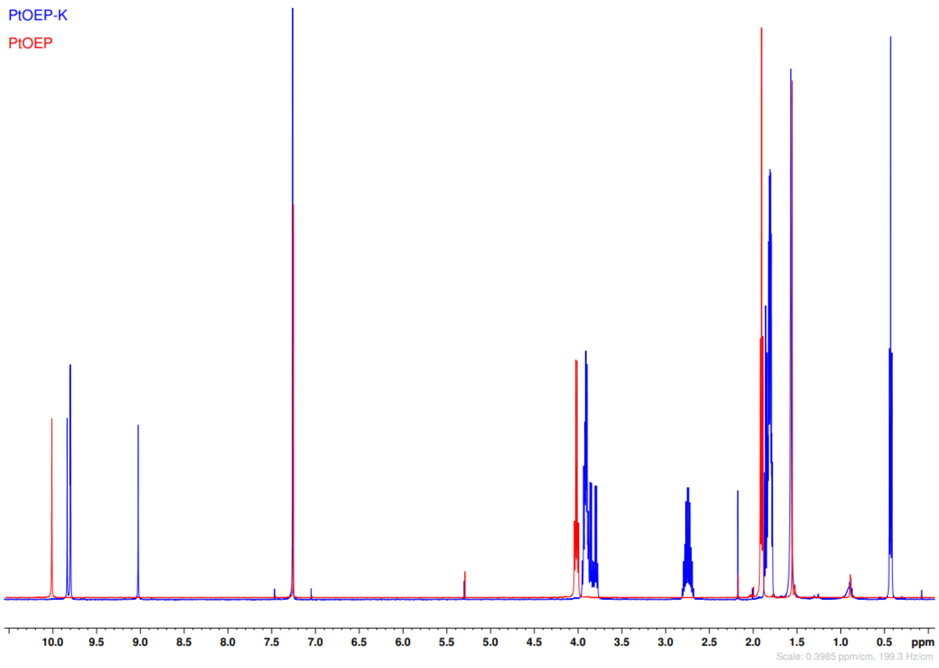

Figure S5. Overlay of ^1H NMR spectra of pure PtOEP (red color) and PtOEP-K (blue color) measured in $CDCl_3$ at 500 MHz.

MALDI-TOF results

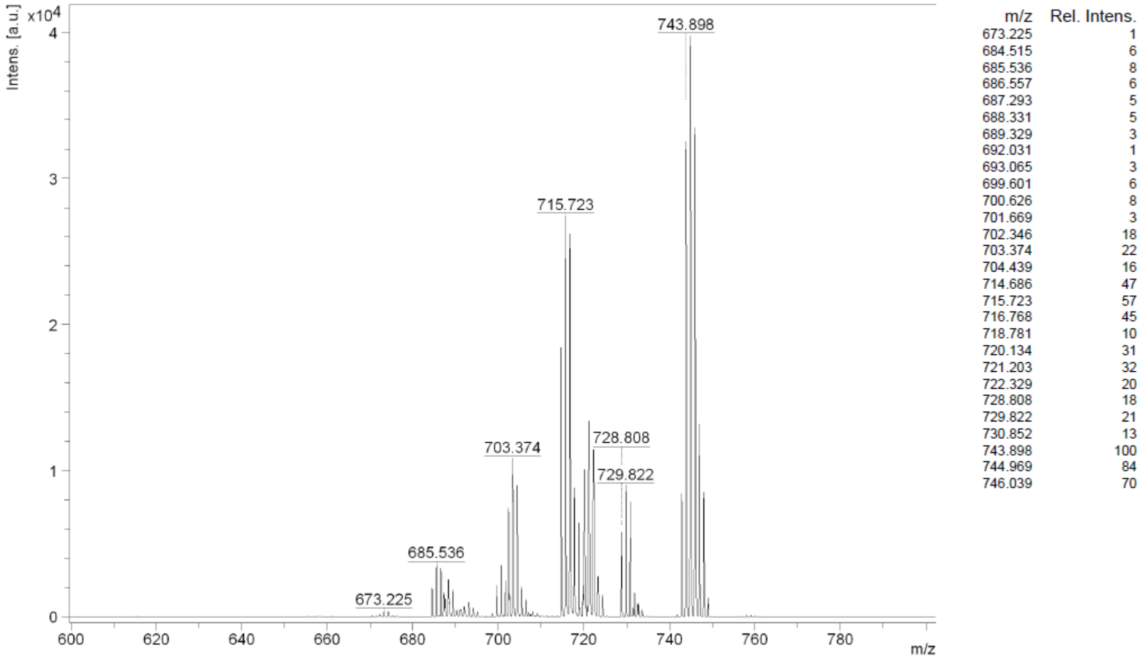

Figure S6. MALDI-TOF (*m/z*) mass spectra for PtOEP-K were recorded on a Bruker Autoflex III Smartbeam instrument: 744 (MH^+, 100%), 730 (21), 721 (32), 716 (57), 703 (22), 686 (8).

TLC results

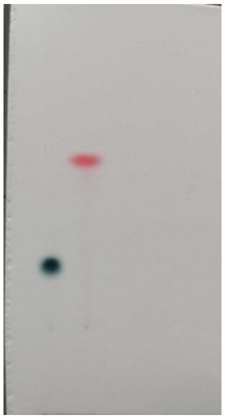

Figure S7. TLC analysis for PtOEP-K (left) and PtOEP (right) when n-hexane/DCM (60:40) is used as mobile phase.

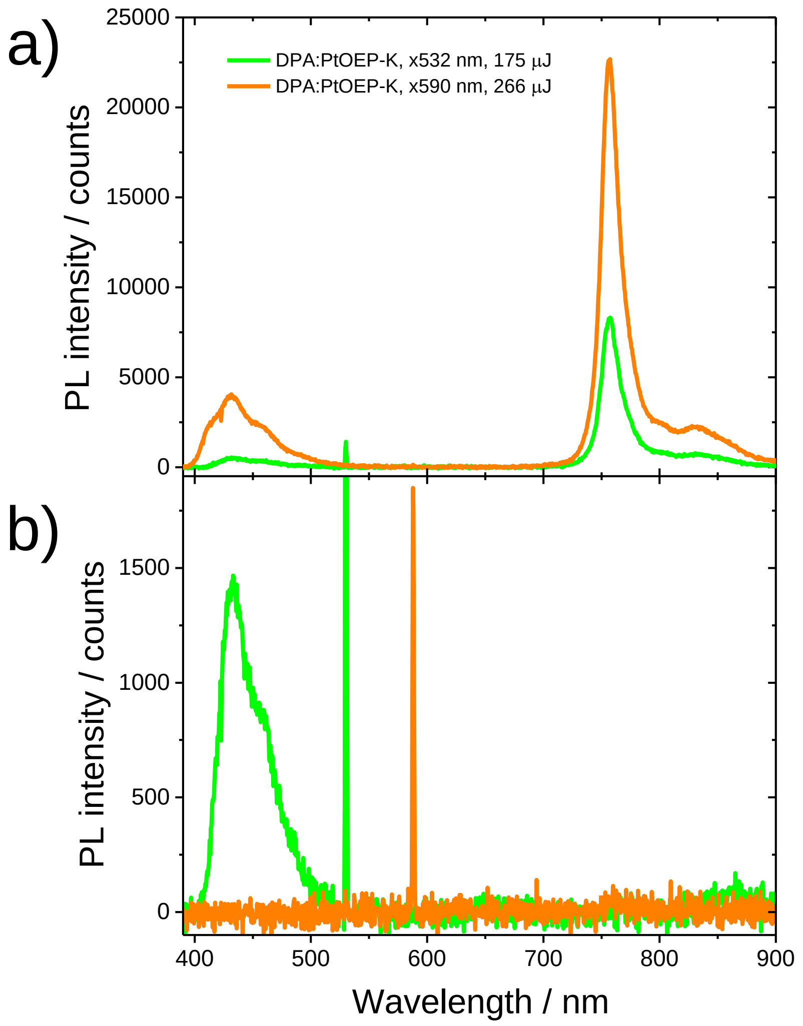

Figure S8. Room temperature, time-integrated PL spectra of a) DPA:PtOEP-K (30 mM:150 μM) and b) DPA:PtOEP (30 mM:150 μM) solutions in degassed toluene after photoexcitation at 532 nm (green line) and 590 nm (orange line). In all cases photoexcitation was with a 10 Hz train of pulses of a Nd:YAG/OPO laser source (6-8 ns laser pulse). All PL spectra were collected with a Newton CCD camera (DU920P-BEX2) coupled to a Czerny-Turner spectrograph (Kymera-328I-B1-SIL, 328 mm focal length, F/4.1 aperture) with an exposure time of 0.25 s after 10 accumulations. Dedicated notch filters were used to suppress the PL intensity of the laser excitation lines; a NF01-532U (Shemrock) for $\lambda_{exc.}$= 532 nm and a ZET594TopNotch (Chroma) for $\lambda_{exc.}$= 590 nm). The averaged laser pulse energy of photoexcitation was determined with a J-10MB-HE pyroelectric sensor coupled to a FieldMax II-TOP power/energy meter (Coherent Inc.)

Rate-equation kinetic model

Figure S9 presents a TTA-UC system comprising an Emitter (E) mixed with a bifunctional annihilator/sensitizer (S) component. In this material combination no triplet energy transfer is energetically probable from triplet-excited Sensitizer to the Emitter and the TTA-UC PL signal of the Emitter is exclusively driven by TTA reactions in the Sensitizer component. Considering a 100% efficient intersystem crossing step (ISC) in the Sensitizer phase, the selective photoexcitation of the Sensitizer in the first singlet excited state S_1 results in the activation of the first triplet excited state, T_1.

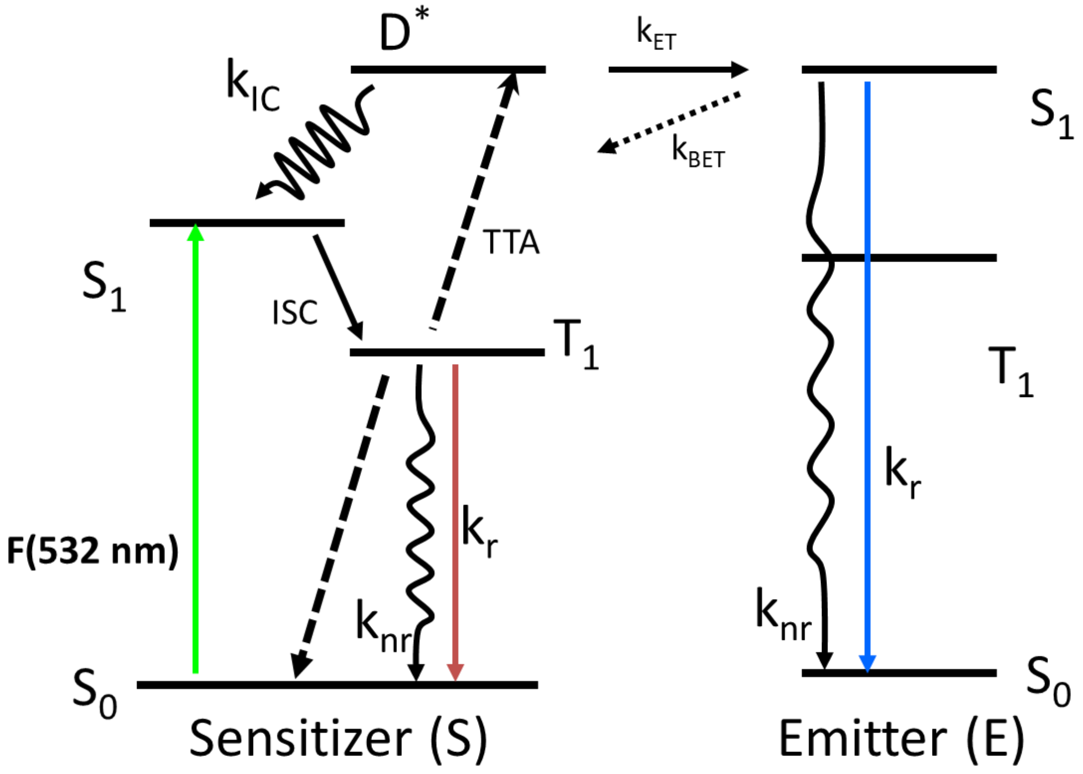

Figure S9. Jablonksi diagram depicting the main photophysical processes that lead to TTA-UC PL activation through a bifunctional sensitizer/activator component.

The deactivation of T_1 in the Sensitizer follows the rate law described by Equation 2.

$$\frac{d[T_1]_S}{dt} = \sigma(\lambda)F(\lambda) - [T_1]_S(k_m^S + k_{TTA}[T_1]_S) \qquad \text{Eq. 2}$$

where $[T_1]_S$ is the triplet-excited state population of Sensitizer component, $\sigma(\lambda)$is the absorption cross-section of the Sensitizer at the photoexcitation wavelength λ, $F(\lambda)$ is the photon flux at wavelength λ, and

k_m^S and k_{TTA} correspond to the apparent monomolecular rate relaxation constant and bimolecular rate constant of the Sensitizer component, respectively. Monomolecular relaxation takes place via both radiative and non-radiative deactivation of the T_1 state, therefore $k_m^S = (k_r^S + k_{nr}^S)$ where k_r^S and k_{nr}^S correspond to the radiative and non-radiative rate constant of the Sensitizer, respectively.

Triplet-triplet annihilation reactions in the Sensitizer component result in the activation of the higher-lying energy state D^* with energy $E_{D^*} \leq 2 \times E_{T_1}$ from where energy can be transferred to the first singlet excited state S_1 of the Emitter component with a rate constant k_{ET}. The rate law dictating the activation and deactivation of the D^* state is described by Equation 3.

$$\frac{d[D^*]_S}{dt} = k_{TTA}[T_1]_S{}^2 - k_q[D^*]_S \qquad \text{Eq. 3}$$

where $[D^*]_S$ corresponds to the population of Sensitizer component in the D^*state. Considering a concentration of Emitter species available to accept the energy from the D^*state of the sensitizer, the apparent monomolecular deactivation rate constant k_q of D^*corresponds to $k_q = k_{IC} + k_{ET} f_1 [S_0]_E$. Here, k_{IC} is the internal conversion deactivation rate of the D^*state and f_1 is the fraction of the ground state S_0 Emitter molecules adjacent to the D^*-excited Sensitizer species with $0 \leq f_1 \leq 1$. The rate law dictating the activation and deactivation of the S_1 state of the Emitter species is described by Equation 4.

$$\frac{d[S_1]_E}{dt} = k_{ET}[D^*]_S - k_m^E [S_1]_E \qquad \text{Eq. 4}$$

Here, k_m^E corresponds to the apparent monomolecular rate constant for the deactivation of S_1 state of the Emitter component with $k_m^E = [(k_r^E + k_{nr}^E) + k_{BET} f_2 [S_0]_S]$, where k_r^E and k_{nr}^E correspond to the radiative and non-radiative rate constant of the Sensitizer, respectively. The fraction $f_2 [S_0]_S$ of the Sensitizer molecules adjacent to the S_1-excited Emitter species, where $0 \leq f_2 \leq 1$, can serve as an energy sink during the resonance back energy transfer process from the S_1 state of the Emitter with a back-energy transfer rate constant k_{BET}.

Under steady-state conditions Equations 2, 3 and 4 become:

$$\sigma(\lambda)F(\lambda) = [T_1]_S (k_m^S + k_{TTA}[T_1]_S) \qquad \text{Eq. 5}$$

$$[D^*]_S = \frac{k_{TTA}}{k_q}[T_1]_S{}^2 \qquad \text{Eq. 6}$$

$$[S_1]_E = \frac{k_{ET}}{k_m^E}[D^*]_S \qquad \text{Eq. 7}$$

Substituting Eq. 6 in Eq.7 one gets:

$$[S_1]_E = \frac{k_{ET}k_{TTA}}{k_q k_m^E}[T_1]_S{}^2 \qquad \text{Eq. 8}$$

The fluence dependence of the sensitizer phosphorescence and the emitter TTA-UC luminescence is described by two major regimes.

i) The apparent monomolecular rate relaxation constant dominates over the bimolecular $k_{TTA}[T_1]_S$ term; that is $k_m^S >> k_{TTA}[T_1]_S$ in Equation 5; the Sensitizer phosphorescence scales linearly with fluence:

$$[T_1]_S = \frac{\sigma(\lambda)}{k_m^S}F(\lambda) \qquad \text{Eq. 9}$$

and the Emitter TTA-UC luminescence exhibits a quadratic dependence on fluence:

$$[S_1]_E = \frac{k_{ET}k_{TTA}}{k_q k_m^E}\left[\frac{\sigma(\lambda)}{k_m^S}\right]^2 F(\lambda)^2 \qquad \text{Eq. 10}$$

ii) The bimolecular $k_{TTA}[T_1]_S$ term overrides apparent monomolecular rate relaxation constant; that is $k_m^S << k_{TTA}[T_1]_S$ in Equation 5; the Sensitizer phosphorescence exhibits a square root dependence on fluence according to Equation 11:

$$[T_1]_S = \frac{\sigma(\lambda)^{1/2}}{k_{TTA}{}^{1/2}}F(\lambda)^{1/2} \qquad \text{Eq. 11}$$

and the Emitter TTA-UC luminescence scales linearly with fluence according to Equation 12.

$$[S_1]_E = \frac{k_{ET}\sigma(\lambda)}{k_q k_m^E}F(\lambda) \qquad \text{Eq. 12}$$

REFERENCES.

(1) Frisch, M. J.; Trucks, G. W.; Schlegel, H. B.; Scuseria, G. E.; Robb, M. A.; Cheeseman, J. R.; Scalmani, G.; Barone, V.; Petersson, G. A.; Nakatsuji, H.; Li, X.; Caricato, M.; Marenich, A. V.; Bloino, J.; Janesko, B. G.; Gomperts, R.; Mennucci, B.; Hratchian, H. P.; Ortiz, J. V.; Izmaylov, A. F.; Sonnenberg, J. L.; Williams-Young, D.; Ding, F.; Lipparini, F.; Egidi, F.; Goings, J.; Peng, B.; Petrone, A.; Henderson, T.; Ranasinghe, D.; Zakrzewski, V. G.; Gao, J.; Rega, N.; Zheng, G.; Liang, W.; Hada, M.; Ehara, M.; Toyota, K.; Fukuda, R.; Hasegawa, J.; Ishida, M.; Nakajima, T.; Honda, Y.; Kitao, O.; Nakai, H.; Vreven, T.; Throssell, K.; Montgomery Jr., J. A.; Peralta, J. E.; Ogliaro, F.; Bearpark, M. J.; Heyd, J. J.; Brothers, E. N.; Kudin, K. N.; Staroverov, V. N.; Keith, T. A.; Kobayashi, R.; Normand, J.; Raghavachari, K.; Rendell, A. P.; Burant, J. C.; Iyengar, S. S.; Tomasi, J.; Cossi, M.; Millam, J. M.; Klene, M.; Adamo, C.; Cammi, R.; Ochterski, J. W.; Martin, R. L.; Morokuma, K.; Farkas, O.; Foresman, J. B.; Fox, D. J. Gaussian 16, Wallingford, CT: 2016.
